# Supplementary material for: Spatial and bulk transcriptomics reveal distinct molecular signatures in Kaposi sarcoma with and without other KSHV-associated diseases
Source: J Transl Med. 2026 Mar 7;24:508. doi: 10.1186/s12967-026-07976-8 (PMC13081650; doi:10.1186/s12967-026-07976-8)
Supplement: Supplementary file 1 — Supplementary material 1 [file 12967_2026_7976_MOESM1_ESM.pdf]

	**KS alone (N=19)**	**KS+KAD (N=23)**	**p-value**
Duration of HIV in years, median (IQR)	13.1 (4.7, 18.9)	7.3 (0.33, 11.7)	0.03
Duration of KS in years, median (IQR)	3.4 (0.9, 10.9)	0.3 (0.07, 4.9)	0.08
Prior KS therapy, n (%)	12 (63)	18 (78)	0.32
HIV VL, copies/ml median (IQR)	20 (<20, 226)	121 (20, 511)	0.10
CD4 T cell count, cells/µl, median (IQR)	211 (87, 538)	80 (32, 193)	0.02
On ART, n (%)	15 (52)	14 (61)	0.32

Supplementary Table 1: Comparisons of HIV and KS characteristics among those with KS alone as compared to those with KS+KAD. Wilcoxon rank-sum tests were used to evaluate differences in continuous variables. Fisher's exact tests were used to compare differences in categorical variables.

Gene Set Enrichment Results

KS + KAD

Pathway	NES	Adjusted P-Value	Core Genes
cytokine activity	−1.95	1.23×10^{-2}	IL32, IL15, CXCL12, IL1RN, TSLP, CXCL3, CXCL16, CX3CL1, CCL4, CXCL14, CCL22, CCL20, IL1B, IL2, IL24, IL34, TNFSF13, WNT4, CSF1, WNT11, WNT10A, CXCL8, WNT5B, CXCL13, FASLG, CCL7, CCL13, CXCL6, CXCL5, CSF2, TNFSF8, WNT2B, CXCL1, CD40LG, TNFSF4, HMGB1, TNFSF9, TNFSF18, IL4, IL1A, WNT3A, CD70, IL11, IFNA1, CSF3, LTB, TNF, WNT2, EDN1
primary metabolic process	1.53	2.54×10^{-2}	MTOR, HIF1A, RRM2, PSMB9, JAK3, CTNNB1, EIF4EBP1, PECAM1, TGFB1, RELN, PDGFA, CCNB1, ITGA1, IRF3, STAT3, IRF2, DLL4, SREBF1, FANCA, BRCA2, PFKFB3, ITGAV, ANGPTL4, UBE2T, JAK1, MLH1, CENPF, DAB2, BATF3, CASP3, BCL6B, ZEB1, ITGB3, MRE11, TNFRSF14, GLUL, KIT, SBNO2, ERCC3, CDH5, FGFR1, FLT1, EGF, TAF3, PSMB5, DUSP5, HELLS, UBE2C, TMEM173, BIRC5, FCGR1A, PSMC4, IL33, RB1, NOTCH1, SPRY4, TBP, APOL6, SIRPA, ENO1, IRF7, IFIH1, OAZ1, DNMT1, PARP9, UBB, KDR, PMS2, CCL21, AKT1, FADD, IFI16, CD40, BLM, MYD88, CD36, DTX3L, TWF1, MARCO, MAP3K12, MMP9, SFRP1, GUSB, CCL2, NF1, TGFB3, GLUD1, TYMS, MGMT, VEGFC, HEY1, MYC, TPI1, TGFBR2, BCAT1, RIPK1, RELA, GZMB, TNFSF12, PIK3CA, TNFRSF11A, TNKS, LILRB2, TNFRSF1B, FZD8, KAT2B, PLOD2, SF3A1, PUM1, THY1, IL10, ITGB2, LDHB, DEPTOR, PDGFRB, STAT1, MB21D1, EGR1, RELB, MSH6, B2M, THBD, PARP4, PSMB10, NFIL3, CDK6, BRCA1, PKM, WNT5A, PIK3CG, IKBKG, FCN1, ABCF1, STAT2, ITGA6, EZH2, CD84, RAD50, IKBKB, HDAC3, PRR5, TNFSF10, EXO1, ROCK1, EIF2AK2, PFKM, TNFRSF1A, EPM2AIP1, THBS1, CD38, BMP2, CSF1R, NFKBIA, POLR2A, GZMH, HDAC5, DTX4, NLRC5, TRIM21, SOCS1, DLL1, ITGB8, CX3CR1, OAS2, CXCL10, C7, SERPINA1, STAT4
natural killer cell activation	−2.00	2.78×10^{-2}	IL2, JAK2, IL18R1, CD2, ULBP2, NCR1, PTPRC, IL21R, IFNA1, CASP8, SLAMF7
B cell proliferation	−1.93	4.47×10^{-2}	CD74, CD40LG, PTPRC, IL4, CD79A, WNT3A, CD70, IFNA1, IL7R, CD19, MS4A1
carbohydrate derivative biosynthetic process	1.99	4.55×10^{-2}	RRM2, JAK3, CTNNB1, TGFB1, EGF, IL33, ENO1, CCL21, TYMS, TPI1

Supplementary Figure 1: Gene Enrichment Analysis differences in pathways between patients with KS and KAD as compared with KS alone per nCounter analyses

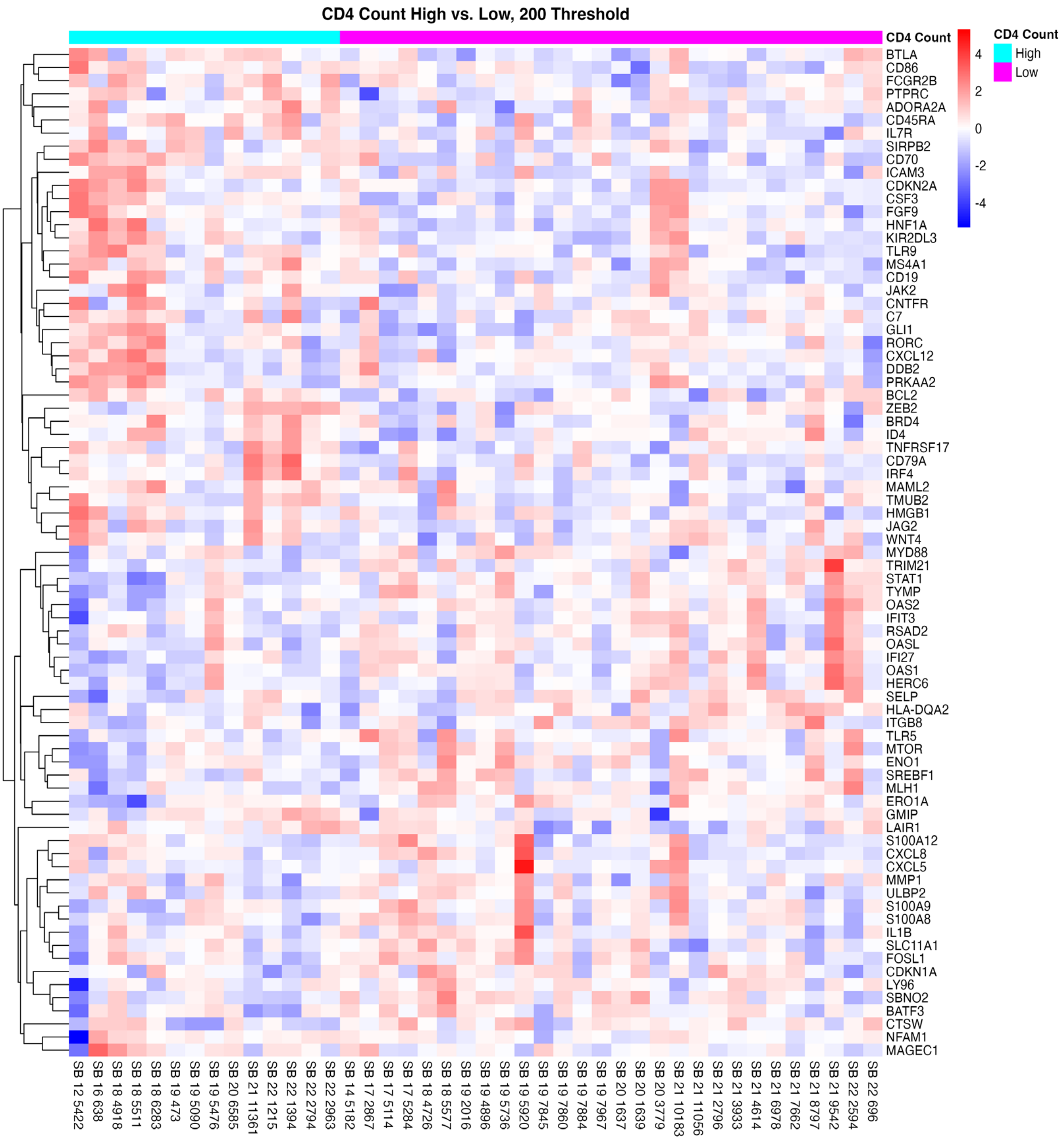

Supplementary Figure 2: Heatmap demonstrating differential gene express profiles by CD4+ T cell count High (>200 cells/µl) vs. Low (<200 cells/µl) using nCounter platform

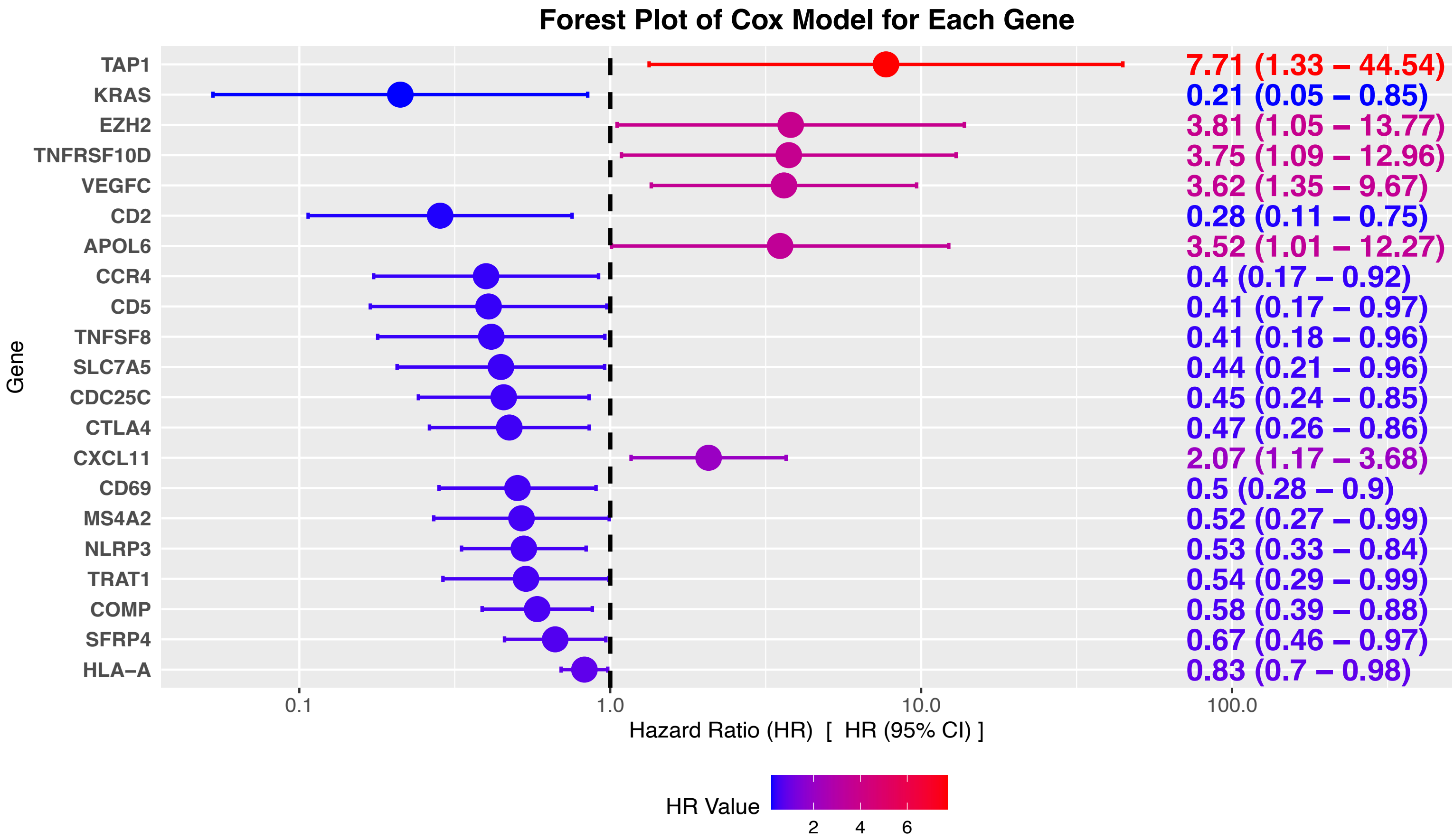

Supplementary Figure 3: Forest plot demonstrating genes from nCounter analyses that were associated with survival outcomes with unadjusted P<0.05
